# TGF-β causes Docetaxel resistance in Prostate Cancer via the induction of Bcl-2 by acetylated KLF5 and Protein Stabilization

**DOI:** 10.7150/thno.44567

**Published:** 2020-06-18

**Authors:** Yixiang Li, Baotong Zhang, Lingwei Xiang, Siyuan Xia, Omer Kucuk, Xingming Deng, Lawrence H. Boise, Jin-Tang Dong

**Affiliations:** 1 Department of Hematology and Medical Oncology, Emory University School of Medicine, Atlanta, GA, 30322, USA.; 2 Department of Radiation Oncology, Emory University School of Medicine, Atlanta, GA, 30322, USA.; 3 Winship Cancer Institute, Emory University, Atlanta, GA, 30322, USA.; 4 ICF, Atlanta, GA, 30322, USA.

**Keywords:** KLF5 acetylation, TGF-β, Bcl-2 degradation, prostate cancer, docetaxel resistance

## Abstract

Prostate cancer is the second leading cause of cancer-related death in the United States. As a first line treatment for hormone-refractory prostate cancer, docetaxel (DTX) treatment leads to suboptimal effect since almost all patients eventually develop DTX resistance. In this study, we investigated whether and how TGF-β affects DTX resistance of prostate cancer.

**Methods:** Cytotoxicity of DTX in DU 145 and PC-3 cells was measured by CCK-8 and Matrigel colony formation assays. Resistance to DTX in DU 145 cells was examined in a xenograft tumorigenesis model. A luciferase reporter system was used to determine transcriptional activities. Gene expression was analyzed by RT-qPCR and Western blotting.

**Results:** We found that KLF5 is indispensable in TGF-β-induced DTX resistance. Moreover, KLF5 acetylation at lysine 369 mediates DTX resistance *in vitro* and *in vivo*. We showed that the TGF-β/acetylated KLF5 signaling axis activates Bcl-2 expression transcriptionally. Furthermore, DTX-induced Bcl-2 degradation depends on a proteasome pathway, and TGF-β inhibits DTX-induced Bcl-2 ubiquitination.

**Conclusion:** Our study demonstrated that the TGF-β-acetylated KLF5-Bcl-2 signaling axis mediates DTX resistance in prostate cancer and blockade of this pathway could provide clinical insights into chemoresistance of prostate cancer.

## Introduction

Prostate cancer is the second leading cause of cancer related deaths in American men, and was expected to cause more than 30,000 deaths in the year 2019 1. Castration-resistant prostate cancer (CRPC) is a lethal form of prostate cancer that has developed resistance to androgen deprivation therapy. Patients with CRPC initially benefit from docetaxel (DTX) as a first line treatment with prolonged survival time and improved rates of response 2, 3. However, most patients receiving DTX ultimately develop resistance 4, 5. Therefore, novel therapeutic strategies are needed to treat DTX-resistant prostate cancer.

DTX induces cytotoxicity in prostate cancer by stabilizing microtubules in actively proliferating cells. It binds to β-tubulin to inhibit the depolymerization of microtubules 6. Extensive research has demonstrated that upregulation of drug efflux pump contributes to DTX resistance and inhibition of multidrug resistance sensitizes cells to chemotherapy 7-9. In addition, several studies have reported that multiple mutations and overexpression of β-tubulin induce DTX resistance by reducing the binding affinity between DTX and β-tubulin 10-12. Epithelial-mesenchymal transition (EMT) has emerged as a critical mechanism mediating DTX resistance by coordinating various intracellular signaling pathways and biological processes, including phosphatidylinositol-3-kinase (PI3K/Akt) signaling and cancer stem cell properties 13-17.

TGF-β activates various cellular processes in human cancer 18-20. Among multiple TGF-β targets, cancer stem cell properties are reported to mediate TGF-β-induced paclitaxel resistance in triple-negative breast cancer 17. Human Krüppel-like factor 5 (KLF5, also known as IKLF or BTEB2) was identified as an indispensable factor for TGF-β induced EMT 21 and an inhibitory factor of DTX induced autophagy 22. The activated TGF-β signaling pathway recruits p300 acetyl-transferase to acetylate KLF5 at lysine 369 and regulates downstream targets with an acetylated KLF5 and Smads complex 23. Moreover, as a dual functional growth factor, TGF-β induces growth inhibition via acetylation on KLF5 lysine 369 in HaCaT cells 24, 25 and prostate cancer cells 26. It is still unclear whether TGF-β-induced KLF5 acetylation is required for DTX resistance of prostate cancer cells.

As an apoptosis suppressor, Bcl-2 dimerizes with pro-apoptotic proteins to sequester mitochondrial outer membrane permeabilization 27. With its pro-survival function, Bcl-2 is indispensable in the transition of prostate cancer cells from androgen-dependence to androgen-independence and correlates with the androgen-independent phenotype 28. Bcl-2 is also reported to mediate chemotherapy resistance in various malignancies including those of the lung, lymphoid, and thyroid 29-31. Moreover, as a result of apoptosis induced by DTX, Bcl-2 was phosphorylated and inactivated 32-34. However, the mechanism by which Bcl-2 mediates TGF-β and acetylated KLF5-induced DTX resistance remains unknown. The present study shows that the KLF5 and TGF-β signaling axis mediates DTX resistance through transcriptionally upregulating Bcl-2 and inhibiting DTX-induced Bcl-2 degradation.

## Material and Methods

### Cell culture and reagents

Human prostate cancer cell lines (DU 145 and PC-3) were obtained from the American Type Culture Collection (ATCC, Manassas, VA, USA), and cultured according to ATCC's instructions. Human recombinant TGF-β1 was purchased from R&D Systems, Inc. (Minneapolis, MN, USA). DTX and SB-505124 were purchased from MilliporeSigma (St. Louis, MO, USA). ABT-199 and S63845 were purchased from Cayman Chemical (Ann Arbor, MI, USA). The jetPRIME transfection reagent (Polyplus transfection, New York, NY, USA) was used for plasmid transfection according to the manufacturer's protocol.

### Endogenous KLF5 knock out cell lines and retroviral expression of KLF5 variants

DU 145 and PC-3 cells with endogenous KLF5 deletion were established as previously described 35. Briefly, the CRISPR-cas9 system was utilized to delete endogenous KLF5 expression following a previous published protocol 26. KLF5-null clones were identified by Western blotting and confirmed by sequencing the PCR product with primers 5'-CACAATCGACAAAATAAGCCTG-3' and 5'-CAGTAGCTGGTACAGGTGGCCC-3'.

In the retroviral system, an expression vector PLHCX with or without different KLF5 variants, pMD2.G and pSPAX2, was transfected into 293T cells with the jetPRIME DNA/siRNA transfection reagent (Polyplus transfection) according to the manufacturer's instructions. Medium containing viruses was filtered at 48 hours (pore size, 0.45 μm, Corning, NY, USA). After virus infection, cells were selected with medium containing hygromycin B (ChemCruz, Dallas, TX, USA) for at least 8 days.

### Cytotoxicity assay

Cytotoxicity was evaluated with water-soluble tetrazolium salt in the Cell Counting Kit-8 (Dojindo Molecular Technologies, Inc., Rockville, MD, USA). Approximately 3×10^3^ cells were seeded in 96-well plates with the indicated TGF-β and/or SB-505124 treatments 24 hours prior to DTX and/or ABT-199 treatment. After 72 hours of DTX treatment, CCK-8 was added to each well, and the plates were incubated for 3 hours, followed by absorbance measurement at 450 nm using the Synergy H1 Hybrid Multi-Mode reader (BioTek, Winooski, VT, USA).

### Colony formation assay

Thick layers of Matrigel matrix (Corning) were used for colony formation assay (3D culture) by adding 50 μl Matrigel to each well of a 96-well plate. After 30 minutes incubation in a 37°C incubator, 200 cells mixed with 100 μl medium containing 5% Matrigel and the indicated TGF-β and/or SB-505124 treatments were seeded in each well. DTX treatment was initiated after 24 hours. The medium was changed every 2 days. After 15 days for DU 145 cells and 7 days for PC-3 cells, colony images were captured using Olympus IX51 Inverted Microscope (Hunt Optics & Imaging, Inc., Pittsburgh, USA) and colonies with diameter greater than 50 μm were counted.

### Xenograft mouse tumorigenesis model

Male BALB/c nude mice (3-4 weeks old) were purchased from Charles River (San Diego, CA), and closely monitored and handled at an Emory University Division of Animal Resources facility according to the policies of the Institutional Animal Care and Use Committee. Equal volumes of PBS and Matrigel were first used to suspend DU 145 cells at 2×10^7^ cells/ml and a 100 µl cell-containing mixture was subcutaneously injected into both flanks of mice. Four mice were used in each group, and DTX was administered daily at a concentration of 1.8 mg/(kg*day) starting from the 7^th^ day. Volumes of eight tumors on four mice were measured every two days, and tumors were surgically isolated on the 23^rd^ day, weighed, and photographed.

### Apoptosis and necrosis assay

Apoptosis and necrosis experiments were conducted with the RealTime-Glo™ Annexin V Apoptosis and Necrosis Assay (Promega, Madison, WI, USA) following the manufacturer's instruction.

Early apoptosis was measured by staining cells with Annexin V-FITC and PI as previously described 36. Briefly, after indicated treatments for 20 hours, cells were collected, washed with cold PBS, resuspended in 100 μl of 1 × Annexin V binding buffer, incubated with 5 μl of Annexin V and PI (BD Pharmingen) for 15 min at room temperature in the dark, and analyzed by flow cytometry. Data was analyzed with FlowJo 7.6 software.

### Western blotting analysis and immunoprecipitation assay

Subconfluent cells were scraped and collected with medium, and washed with cold phosphate-buffered saline (PBS), harvested with 2× Laemmli Sample Buffer (Bio-Rad, Hercules, CA, USA) and heated at 98 °C for 5 minutes. Whole cell lysates were separated by SDS-PAGE and blotted onto nitrocellulose membranes (Bio-Rad). Membranes were blocked with 5% non-fat milk in a TBS buffer (PH 8.0) with 0.1% Tween-20, and further incubated overnight at 4 °C with primary antibody (Bcl-2 antibody: 15071, Bax antibody: 2772, Bak antibody: 12105, Bcl-xL antibody: 2764, PARP antibody: 9542, Cell Signaling Technology, Danvers, MA, USA. Β-actin antibody: sc-47778, Santa Cruz Biotechnology, Dallas, TX, USA. Mcl-1 antibody: AB2910, MilliporeSigma, St. Louis, MO, USA). Membranes were rinsed 3 × 5 minutes with TBS buffer (PH 8.0) with 0.1% Tween-20 and incubated with secondary antibody (anti-rabbit IgG, HRP-linked antibody: 7074, anti-mouse IgG, HRP-linked antibody: 7076, Cell Signaling Technology) for 2 hours at room temperature. Membranes were then washed for 3 x 5 minutes in TBS buffer (PH 8.0) with 0.1% Tween-20 followed by visualization using the ECL detection system and LAS-4000 (GE Healthcare Buckinghamshire, UK).

In the immunoprecipitation assay, whole cell lysates were collected by incubating 4 hours with RIPA buffer (MilliporeSigma) supplemented with 0.5% Protease Inhibitor Cocktail (Sigma), and centrifuged at 12,000 rpm for 2 minutes at 4 °C. Cell lysate, the supernatant, was incubated with HA-Tag antibody (3724; Cell Signaling Technology) or KLF5 antibody (AF 3758; R&D Systems) overnight at 4 °C. Protein and antibody were then incubated with Protein G Sepharose beads (MilliporeSigma) for 2 hours at 4 °C. Samples were rinsed three times with 500 μl RIPA buffer and then centrifuged at 1,800 rpm at 4 °C. Protein G pellets were suspended with 2× Laemmli Sample Buffer (Bio-Rad) and heated at 98 °C for 5 minutes.

### Luciferase reporter system

*BCL2* promoter LB322 plasmid (Bcl-2 from ATG to -3934) was a gift from Linda Boxer (Addgene plasmid # 15381; http://n2t.net/addgene:15381; RRID: Addgene_15381) 37. The jetPRIME transfection reagent (Polyplus transfection) was used for plasmid transfection according to the manufacturer's protocol. Forty-eight hours after transfection, cells were lysed with 100 µl of Passive Lysis Buffer (Promega, Madison, WI, USA), and luciferase activities were measured with the Luciferase Assay System (Promega) on a Berthold FB12 Luminometer (Berthold, Bad Wildbad, Germany). Luciferase activities were normalized by protein concentration measured by Pierce^TM^ BCA Protein Assay Kit (Thermo Fisher Scientific, Waltham, MA, USA). Each data point was repeated in triplicate.

### Realtime qPCR

Trizol reagent (Invitrogen, Carlsbad, CA, USA) was used to isolate total RNA. First strand cDNA was synthesized from total RNA with the RT-PCR kit from Promega. Realtime qPCR with SYBR Green master mix was used to measure *BCL2* mRNA level in the Applied Biosystems^TM^ 7500 Real-Time PCR System (Thermo Fisher Scientific). Primer sequences for *BCL2:* Forward: 5'-GAGAAATCAAACAGAGGCCG-3', Reverse: 5'-CTGAGTACCTGAACCGGCA-3'.

### DTX resistant cell lines

DTX resistant cell lines were established following a dose escalation strategy. DU 145 parental cells were initially cultured in medium containing 0.5 nM DTX, and then the cells were subcultured every two weeks in medium with a 50% increase in DTX concentration. The resulting DTX-resistant cell lines tolerated a final DTX concentration of 50 nM (DDR50) and 100 nM (DDR100).

### Statistical analysis

Data was expressed as means ± standard errors of the mean (SEM). The statistical significance of the difference in means of two groups was determined with two tailed unpaired Student *t* test. Two-way Anova test was used to analyze mouse xenograft tumor volume curves. *P*-values greater than 0.05 were considered statistically insignificant (ns), *p*-values less than or equal to 0.05 were considered statistically significant (*). *P-*values less than or equal to 0.01 and 0.001 were labeled as ** and ***, respectively.

## Results

### KLF5 is required by TGF-β to induce DTX resistance in prostate cancer cells

**Figure 1:**
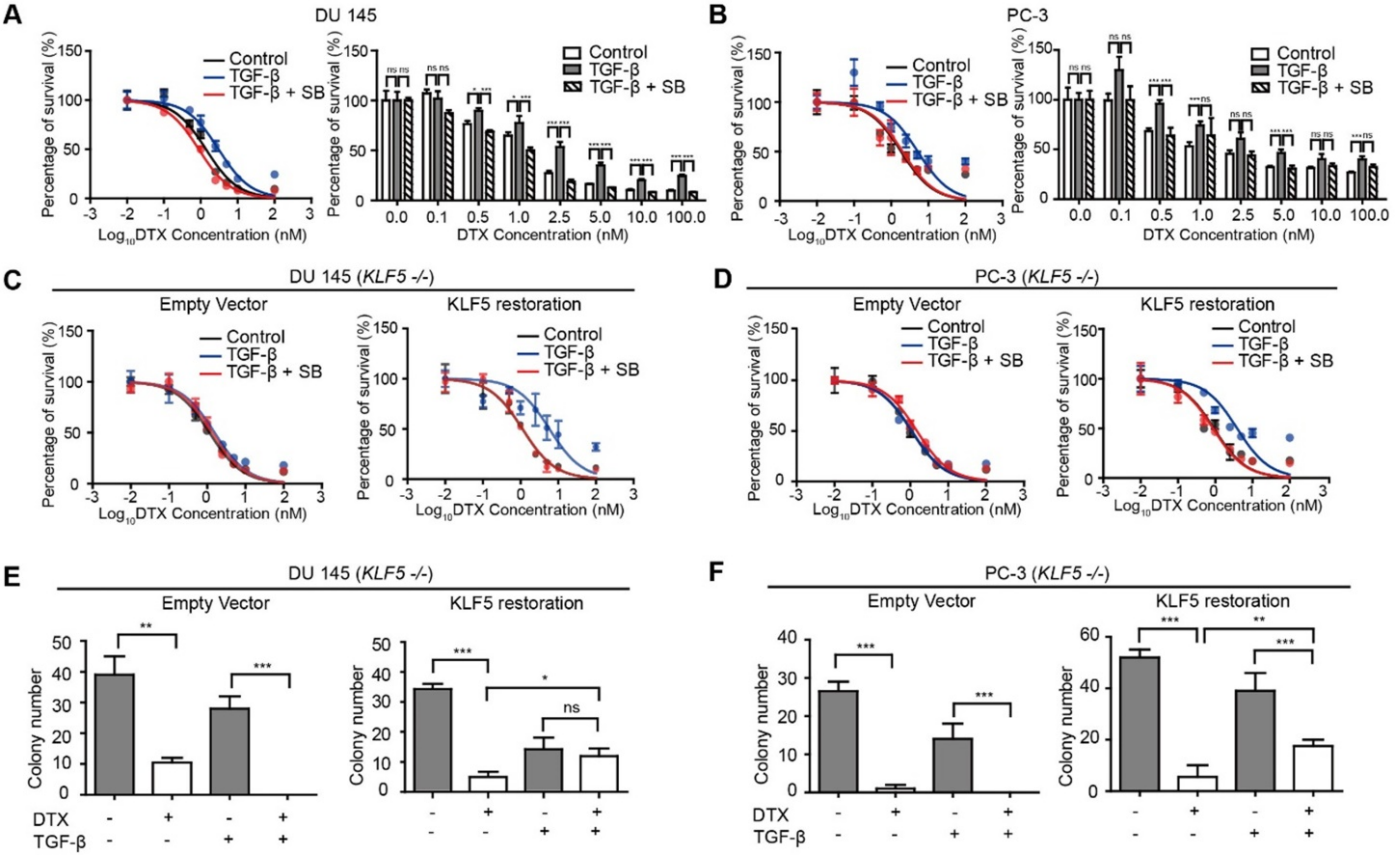
** KLF5 is required by TGF-β to induce DTX resistance in prostate cancer cells.** (A, B) Cytotoxicity assay in prostate cancer DU 145 and PC-3 cells with concomitant treatment with docetaxel (DTX) and TGF-β1 (10 ng/µl) and/or TGF-β receptor I inhibitor, SB505124 (SB, 2.5 µM). (C, D) Cytotoxicity assay in DU 145 and PC-3 cell variants with concomitant treatment with DTX and TGF-β1 (10 ng/µl) and/or SB505124 (2.5 µM). *KLF5 -/-*, endogenous *KLF5* knockout. (E, F) Colony formation assay of *KLF5* -/- DU 145 and PC-3 cells with or without wild type *KLF5* restoration in Matrigel treated with DTX (1 nM) and/or TGF-β1 (10 ng/µl). Cytotoxicity assay and Matrigel colony formation assay were performed in triplicate, and error bars represent the standard errors of the means. ns, *p* > 0.05; *, *p* ≤ 0.05; **, *p* ≤ 0.01; ***, *p* ≤ 0.001. DTX: docetaxel; SB: SB-505124.

Several previous studies reported that TGF-β induces DTX resistance 17, 38, 39. In order to measure the level of DTX resistance of prostate cancer cell lines DU 145 and PC-3, we conducted *in vitro* cell survival assays following treatment with DTX and TGF-β. We found that treatment with 10 ng/μl TGF-β led to a two-fold increase in DTX IC_50_ (from 1.13 to 2.40 nM) in DU145 prostate cancer cells. Specifically, starting at 2.5 nM DTX treatment, cell survival was more than 2-fold greater in cells treated with 10 ng/μl TGF-β (*p*-value < 0.001), while TGF-β inhibition with 2.5 μM SB-505124 led to a decrease in cell survival of more than 50% (*p*-value < 0.05), with an IC_50_ of 0.96 nM (Figure 1A). Moreover, increasing the concentration of DTX led to a greater difference in the percentage of cells surviving (Figure 1A, right panel). At a DTX concentration lower than 1 nM, the percentage of cell survival after treatment with TGF-β and SB-505124 was similar, while with DTX concentrations higher than 1 nM, we began to observe significant differences in cell survival (Fig 1A). Similar trends were observed in PC-3 cells. Treatment with 10 ng/μl TGF-β induced a three-fold increase in IC_50_ (from 1.87 nM to 5.22 nM) and higher cell survival percentages at DTX concentrations of 0.5, 1, 5, and 100 nM (p-value<0.001). On the other hand, the IC_50_ decreased from 5.22 nM to 2.03 nM in cells treated with the combination of TGF-β and SB-505124 (Figure 1B). These results further showed that TGF-β induced DTX resistance and inhibition of TGF-β signaling by SB-505124 sensitized cells to DTX treatment.

Next, we explored whether TGF-β-induced DTX resistance depends on KLF5 expression. We first knocked out *KLF5* endogenously using the CRISPR-Cas9 system in DU 145 and PC-3 cells and measured the DTX sensitivity of these cells with an *in vitro* cell survival assay. Interestingly, TGF-β or SB-505124 treatment did not change the DTX sensitivity when KLF5 was endogenously knocked out (Figure 1C-D, S1A-B left panels). In contrast, after restoration of KLF5 expression in DU 145 and PC-3 *KLF5*-null cells, TGF-β treatment alone increased DTX resistance, with an increase in IC_50_ from 1.1 to 5.8 nM in DU 145 cells, and from 0.9 to 3.9 nM in PC-3 cells (Figure 1C-D, S1A-B right panels). In cells treated with the combination of SB-505124 and TGF-β, IC_50_ values were 1.1 nM in DU 145 cells and 0.88 nM in PC-3 cells, which were similar to those of the control group. Notably, cell survival was more than 2-fold greater after 2.5, 5, 10, and 100 nM DTX treatment in the TGF-β treatment group than in the control group or the TGF-β and SB505124 combination treatment group (Figure S1A-B).

Colony formation assay in 3D Matrigel was used to simulate an *in vivo* environment with DTX treatment. After 1 nM DTX treatment for 15 days, colonies with diameters greater than 50 μm were counted. DTX treatment effectively inhibited growth and killed *KLF5*-null cells regardless of TGF-β treatment (Figure 1E-F left panels, S1C-D). Furthermore, KLF5 restoration in DU 145 and PC-3 *KLF5*-null cells contributed to TGF-β-induced DTX resistance. In DU 145 KLF5 -/- cells with restored wild-type KLF5, treatment with DTX in the absence of TGF-β inhibited growth and caused cell death, but in the presence of TGF-β DTX did not reduce colony numbers significantly (*p*-values > 0.05) (Figure 1E right panel, S1C), although TGF-β decreased colony numbers. In PC-3 KLF5 -/- cells with restored wild-type KLF5, although DTX treatment significantly reduced colony numbers in the presence and absence of TGF-β, this effect was greater in the absence of TGF-β (*p*-value < 0.01) (Figure 1F right panel, S1D). Using *in vitro* cytotoxicity assay and three-dimensional colony formation assay with Matrigel, we concluded that KLF5 is essential for TGF-β to induce DTX resistance in DU 145 and PC-3 cells.

### KLF5 acetylation mediates TGF-β induced DTX resistance

**Figure 2:**
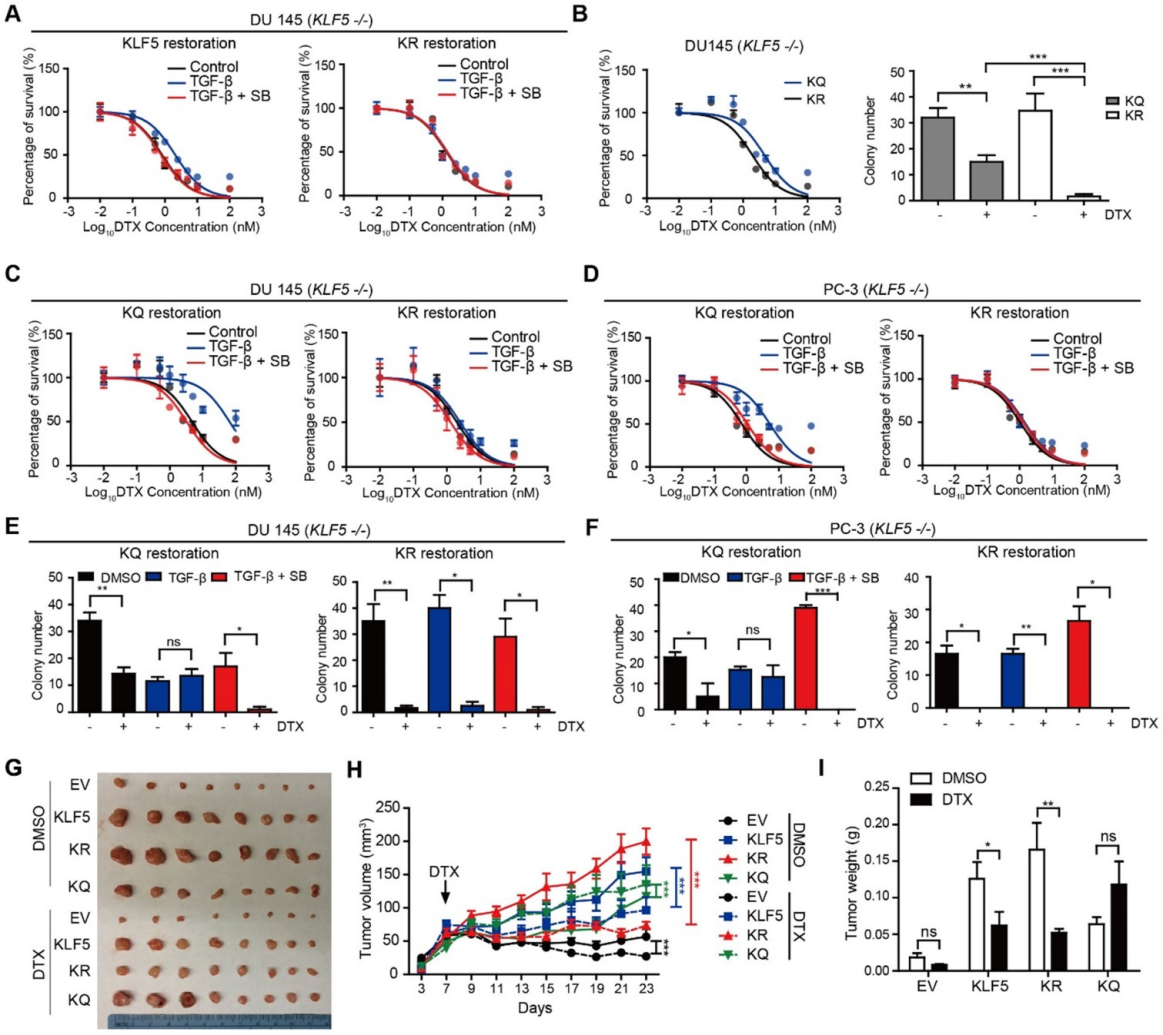
** KLF5 acetylation at K369 mediates DTX resistance in prostate cancer cells.** (A) Cytotoxicity assay in DU 145 (*KLF5*-/-) cells expressing wild type *KLF5* and acetylation deficient mutant KLF5^K369R^ (KR) with concomitant treatment with DTX and TGF-β1 (10 ng/µl) and/or SB505124 (2.5 µM). (B) Cytotoxicity assay (left) and colony formation assay in Matrigel (right) of DU 145 (KLF5-/-) cells expressing acetylation deficient mutant KLF5^K369R^ (KR) and acetylation mimicking mutant KLF5^K369Q^ (KQ) treated with DTX (1 nM). (C - F) Cytotoxicity assay of DTX (C, D) and colony formation assay with 1 nM DTX (E, F) in DU 145 and PC-3 (KLF5-/-) cells expressing KR and KQ with concomitant treatment of TGF-β1 (10 ng/µl) and/or SB505124 (2.5 µM). (G - I) Xenograft tumorigenesis assay with DU 145 KLF5 -/- (EV), wild-type KLF5 (KLF5), KR, KQ cells. Eight tumors from 4 nude mice were available for each group. Cytotoxicity assay and Matrigel colony formation assay were performed in triplicate, and error bars represent the standard errors of the means. ns, *p* > 0.05; *, *p* ≤ 0.05; **, *p* ≤ 0.01; ***, *p* ≤ 0.001. DTX: docetaxel; SB: SB-505124.

Previously, we found that TGF-β induced KLF5 acetylation at lysine 369 by recruiting P300 acetyltransferase. Therefore, we wanted to know if KLF5 lysine 369 mediates TGF-β-induced DTX resistance. We used the lentivirus system to restore wild-type KLF5 and acetylation deficient mutant KLF5^K369R^ (KR) in *KLF5*-null DU 145 cells before treatment with TGF-β and/or its inhibitor. We found that point mutation at KLF5 lysine 369 blocked TGF-β-induced DTX resistance (Figure 2A). In addition, we wondered if acetylated KLF5 mediates TGF-β-induced DTX resistance. Using the lentivirus system, we restored acetylation deficient mutant KLF5^K369R^ (KR) and acetylation mimicking mutant KLF5^K369Q^ (KQ) in KLF5 -/- DU 145 and PC-3 cells. The IC_50_ of DTX was more than 2-fold greater in cells expressing the KQ mutant than in cells expressing the KR mutant (IC_50_ 4.7 vs. 2.0 nM, respectively) (Figure 2B left, S2B). KQ expression led to more than 20% increase in cell survival percentage starting from 1 nM DTX concentration. In addition, we performed a 3D colony formation assay with Matrigel for 15 days. We found that, although DTX significantly decreased colony numbers in both KQ and KR cells, cells expressing KQ had greater colony formation than those expressing KR with 1 nM DTX treatment (Figure 2B right). These results suggest that KLF5 acetylation at K369 induced DTX resistance in DU 145 cells.

We next studied whether TGF-β-induced DTX resistance depends on acetylated KLF5. *In vitro*, 72 hours of 10 ng/μl TGF-β treatment induced a 5-fold increase in DTX IC_50_ (from 3.15 nM to 15.04 nM) in DU 145 KQ cells and more than 7-fold increase (from 0.72 nM to 5.29 nM) in PC-3 KQ cells (Figure 2C-D, S2D-E left panels). Moreover, TGF-β inhibition by SB-505124 successfully eliminated the increase in IC_50_ induced by TGF-β treatment in both DU 145 and PC-3 cells (Figure 2C-D, S2D-E right panels). Colony formation assay in Matrigel was performed to measure DTX resistance in a three-dimensional environment (Figure 2E-F, S2F-G left panels). In control DU 145 and PC-3 KQ cells, treatment with 1 nM DTX reduced colony numbers by more than 50%. While TGF-β demonstrated a growth inhibitory effect, DTX did not inhibit colony formation of KQ cells in the presence of TGF-β. Inhibition of TGF-β by its receptor inhibitor SB-505124 sensitized KQ cells to DTX, as shown by a drastic decrease in the number of colonies surviving DTX treatment. Additionally, we assessed whether TGF-β induces DTX resistance in acetylation deficient mutant KLF5^K369R^ (KR) cells. Interestingly, in both DU 145 and PC-3 KR cells, TGF-β and its inhibition failed to change IC50 significantly (Figure 2C-D, S2D-E right panels). Moreover, in the colony formation assay, TGF-β and its inhibition did not affect colony survival under DTX treatment (Figure 2E-F, S2F-G right panels). These results show that TGF-β requires acetylated KLF5 to induce DTX resistance.

### Acetylated KLF5 induced DTX resistance *in vivo*

Next, we conducted xenograft mouse tumorigenesis assays to explore the function of acetylated KLF5 in a pre-clinical setting. DTX was administered to nude mice 7 days after subcutaneous injection of DU 145 empty vector (EV), KLF5, KR, and KQ cells in the context of KLF5 knockout as we described above. The EV and KQ groups had smaller tumor burden than the KLF5 and KR groups in the absence of DTX treatment. Consistent with a previous report 26, this suggests wild type KLF5 and acetylation deficient KLF5 promote tumor growth while acetylated KLF5 inhibits tumor growth. In DTX treated mice, we observed the largest reduction in tumor burden (tumor volume and weight) in the KR group, and no significant reduction in the KQ group (Figure 2G - I). Consistent with the *in vitro* study, data from the xenograft mouse model suggests that KLF5 acetylation induces DTX resistance while acetylated deficient KLF5 sensitizes cells to DTX treatment.

### Acetylated KLF5 upregulates Bcl-2 expression transcriptionally and inhibition of Bcl-2 sensitizes cells to DTX

**Figure 3:**
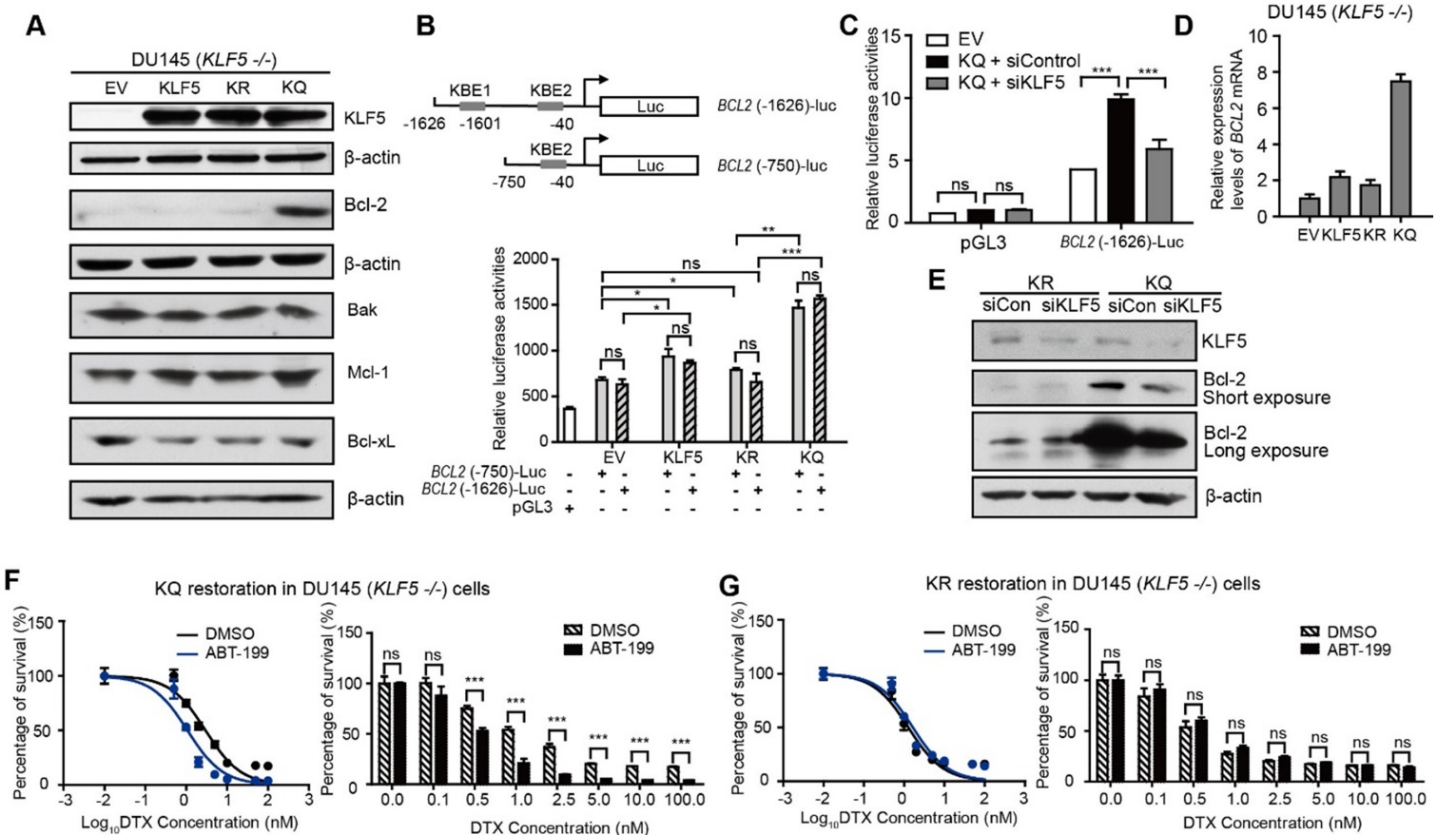
** Acetylated KLF5 induces DTX resistance by upregulating Bcl-2.** (A) Western blotting analysis of Bcl-2 family proteins in isogenic *KLF5* null (*KLF5* -/-) DU 145 cells expressing empty vector (EV), KLF5^WT^ (KLF5), KLF5^K369R^ (KR), and KLF5^K369Q^ (KQ). β-actin is used as endogenous control. (B) Mapping of promoter of *BCL2* mRNA regulated by acetylated KLF5 by transfecting *BCL2* promoter truncations with pGL3 plasmid backbone in DU 145 EV, KLF5, KR, KQ cells. (C) Relative luciferase activities in EV, KQ and KQ cells transfected with *KLF5* siRNA. (D) Relative mRNA levels of *BCL2* in DU 145 EV, KLF5, KR, and KQ cells, as detected by real-time qPCR with GAPDH as endogenous control. (E) Detection of Bcl-2 and β-actin (endogenous control) proteins by Western blotting after *KLF5* knockdown by siRNA in DU 145 KR and KQ cells. (F, G) Cytotoxicity assay of DTX in DU 145 KQ and KR cells treated with Bcl-2 inhibitor, ABT-199 (500 nM). Real time qPCR assay, and cytotoxicity assay were performed in triplicate, and error bars represent the standard errors of the means. ns, *p* > 0.05; *, *p* ≤ 0.05; **, *p* ≤ 0.01; ***, *p* ≤ 0.001. DTX: docetaxel.

Next, we aimed to explore the mechanism of DTX resistance mediated by acetylated KLF5 and TGF-β in prostate cancer cells. First, we tested if DTX caused DU 145 cell death through an apoptotic pathway. As an early indicator of apoptosis, the level of phosphatidylserine (PS) was measured using the RealTime-Glo Annexin V Apoptosis and Necrosis Assay. In DU 145 cells, 10 nM DTX treatment induced a sharp increase in Annexin V signal after 24 hours of treatment as detected by luminescence; however, cell permeability was not elevated until 36 hours (Figure S3A). This shows that, rather than PS exposure and cell permeability occurring at the same time, PS exposure was observed 12 hours before cell membrane breakdown. This further indicates that DTX is an apoptosis inducer. Second, with the same assay, we found that de-acetylated KLF5 mutant induced a 1.5-fold greater level of Annexin V signal starting from 24 hours of DTX treatment (Figure S3B). This indicates that DTX treatment induced a stronger early apoptosis response in KR cells than in KQ cells. To identify the molecule mediating this early apoptosis event, Bcl-2, Bak, Mcl-1, Bcl-xL, Bax levels were measured in cells with different KLF5 acetylation status via Western blotting. Isogenic DU 145 *KLF5*-null cells restored with EV, wild type KLF5, KR, and KQ had similar expression levels of Bak and Bcl-xL, while none of them expressed Bax, as confirmed by previous reports of Bax missense mutation in DU 145 cells 40. Interestingly, KQ cells had a significantly higher level of Bcl-2, while KLF5 and KQ cells had slightly increased Mcl-1 levels (Figure 3A).

We used a luciferase reporter system to test if acetylated KLF5 regulates Bcl-2 at the transcriptional level. We found two predicted KLF5 binding elements (KBE) in the Bcl-2 promoter region at -1601~-1611 with sequence: *CCCCTCCGCCC* and -40~-50 with sequence: *GCTCCCACCCC* 41, 42. Truncation mutants (KBE1: *BCL2* (-1626)-Luc and KBE2: *BCL2* (-750)-Luc) were constructed before the firefly luciferase sequence to measure relative luminance in DU 145 cells (Figure 3B). DU 145 KLF5 null cells were first overexpressed with EV, wild type KLF5, KR or KQ. The relative luminance in the KLF5 group was 1.2-fold greater than that in the EV group (*p-*values < 0.05 comparing both truncation mutants separately). Compared to the EV group, the KR group showed similar luminance (*p-*value < 0.05 with *BCL2* (-1626)-Luc, p value>0.05 with *BCL2* (-750)-Luc), while the KQ group showed an increase in luminance of more than 2-fold (*p*-values < 0.0001 comparing both truncation mutants separately). The two truncation mutants showed similar luminance, indicating that KBE1 is not the potential acetylated KLF5 regulating region. Furthermore, we knocked down KLF5 with *KLF5* siRNA and found decreased luminance level to 50% of the siRNA control group (Figure 3C). To test if Bcl-2 mRNA levels were increased, we conducted quantitative RT-PCR. We found that relative *BCL2* mRNA expression was increased by 8-fold compared to the vehicle control group and more than 4-fold compared to the wild-type KLF5 and de-acetylated KLF5 mimic groups (Figure 3D). In addition, as detected by Western Blotting, knockdown of *KLF5* in KQ cells downregulated Bcl-2 protein level, but did not affect that in KR cells (Figure 3E). Therefore, as a transcriptional factor, acetylated KLF5 upregulates Bcl-2 transcriptionally.

Next, we assess the ability of a small molecule Bcl-2 specific inhibitor, ABT-199, to sensitize prostate cancer cells to DTX treatment. Interestingly, treatment with 500 nM ABT-199 sensitized KQ cells to DTX treatment. A significantly smaller percentage of cells survived DTX treatment, and the IC_50_ decreased by 30% (from 3.0 nM to 1.1 nM) after ABT-199 treatment combined with DTX (Figure 3F). However, treatment with 500 nM ABT-199 failed to sensitize KR cells to DTX treatment (Figure 3G). Thus, Bcl-2 inhibition by ABT-199 successfully inhibits DTX resistance induced by acetylated KLF5.

In addition, we used a Mcl-1 specific inhibitor, S63845, to treat KR and KQ cells in the DTX cytotoxicity assay. The concentration of S63845 selected was the maximum that cells could tolerate. We found that S63845 did not significantly shift the survival curve in either KR or KQ cells (Figure S3C-D), suggesting that Mcl-1 does not play a significant role in TGF-β induced DTX resistance in KQ cells.

### TGF-β induces KLF5 acetylation and Bcl-2 expression

**Figure 4:**
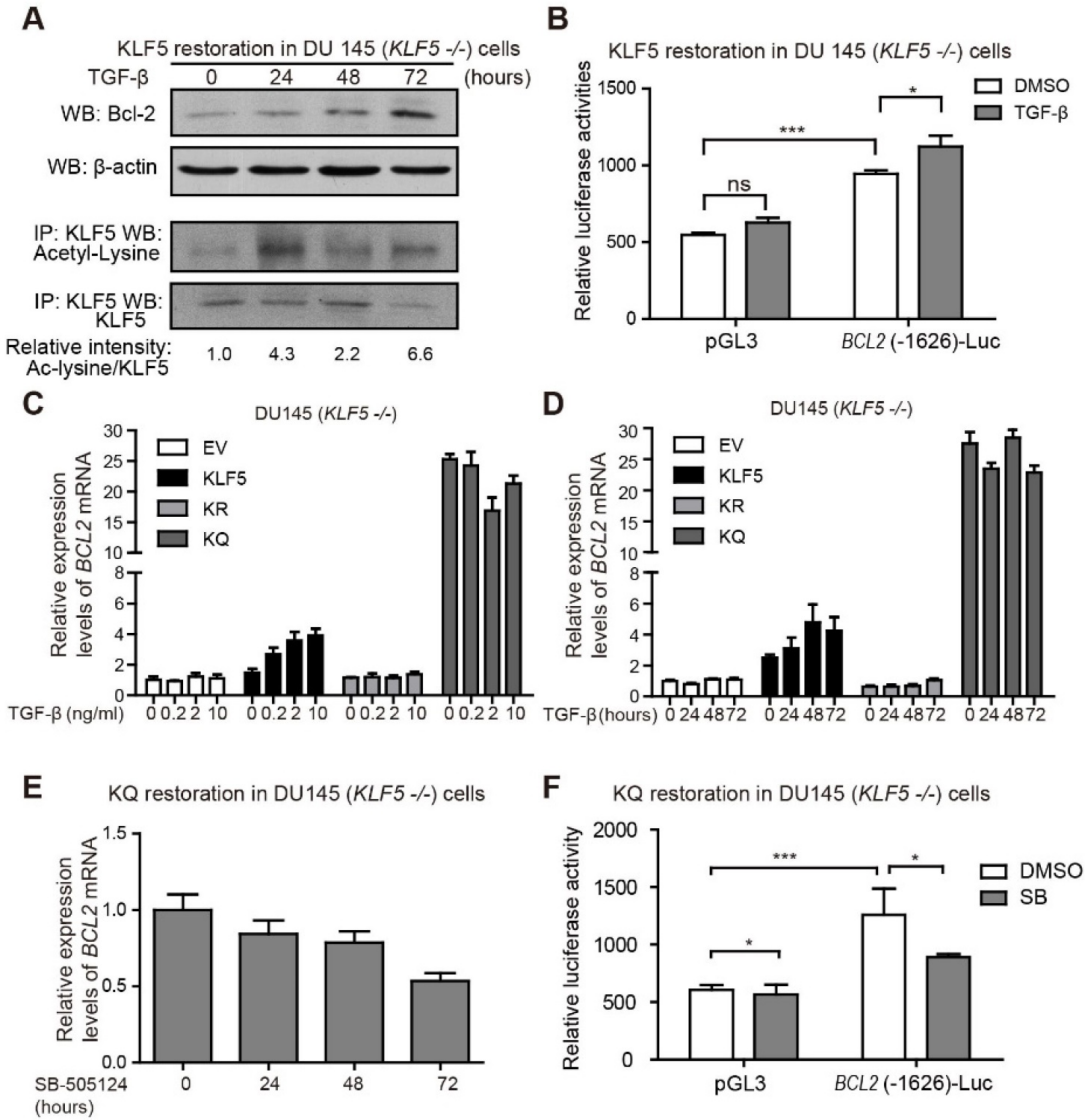
** TGF-β induces KLF5 acetylation and upregulates Bcl-2 expression.** (A) Western blotting analyses of KLF5 and Bcl-2 in whole cell protein lysates and acetylated lysine in KLF5 immunoprecipitated protein lysates, after 0, 24, 48, 72 hours treatment with 10 ng/ml TGF-β1. (B) Relative luciferase activities in KLF5 cells transfected with *BCL2* promoter and treated with TGF-β1. (C, D) Relative mRNA levels of *BCL2* in DU 145 EV, KLF5, KR, and KQ cells treated with 0, 0.2, 2, 10 ng/µl TGF-β1 for 48 hours (C), TGF-β1 (10 ng/µl) for 0, 24, 48, 72 hours (D), as detected by real-time qPCR with GAPDH as endogenous control. (E) Relative mRNA level of *BCL2* in DU 145 KQ cells treated with 2.5 nM SB-505124 for 0, 24, 48, 72 hours, as detected by real-time qPCR with GAPDH as endogenous control. (F) Relative luciferase activities in KQ cells transfected with *BCL2* promoter and treated with SB-505124 (SB, 2.5 µM). Real-time qPCR assay, and luciferase activity assay were performed in triplicate, and error bars represent the standard errors of the means. ns, *p* > 0.05; *, *p* ≤ 0.05; **, *p* ≤ 0.01; ***, *p* ≤ 0.001.

Next, we further explored the role of TGF-β in regulating Bcl-2 expression. DU 145 KLF5 null cells restored with wild-type KLF5 were treated with TGF-β1 for 72 hours. Protein samples were harvested at 0, 24, 48, and 72 hours after treatment. As shown by Western blotting, TGF-β induced upregulation of acetylated KLF5 as early as 24 hours, and Bcl-2 expression starting from 48 hours (Figure 4A). Moreover, luciferase reporter assay showed an increase in relative luciferase activity in cells restored with wild-type KLF5 after transfection of *BCL2*(-1626)-Luc, which was further augmented by TGF-β treatment (Figure 4B). Real-time qPCR assay showed that TGF-β treatment increased *BCL2* mRNA level in KLF5 cells but not in EV, KR, or KQ cells (Figure 4C-D). These results indicate that induction of KLF5 acetylation by TGF-β is essential for TGF-β to induce *BCL2* mRNA. In cells with *KLF5* null or KLF5 acetylation deficient mutant, TGF-β failed to initiate *BCL2* transcription. As a TGF-β activated form of KLF5 mimic, KQ cells did not respond to TGF-β treatment. However, TGF-β inhibition by SB-505124 decreased *BCL2* mRNA level in KQ cells and decreased luciferase activity in KQ cells transfected with *BCL2*(-1626)-Luc, suggesting that TGF-β signaling is indispensable in the transcriptional activation of *BCL2* by acetylated KLF5 (Figure 4E-F). These results further showed that TGF-β induces KLF5 acetylation, and acetylated KLF5 activated *BCL2* expression depends on TGF-β signaling.

### TGF-β inhibits Bcl-2 degradation induced by DTX

**Figure 5:**
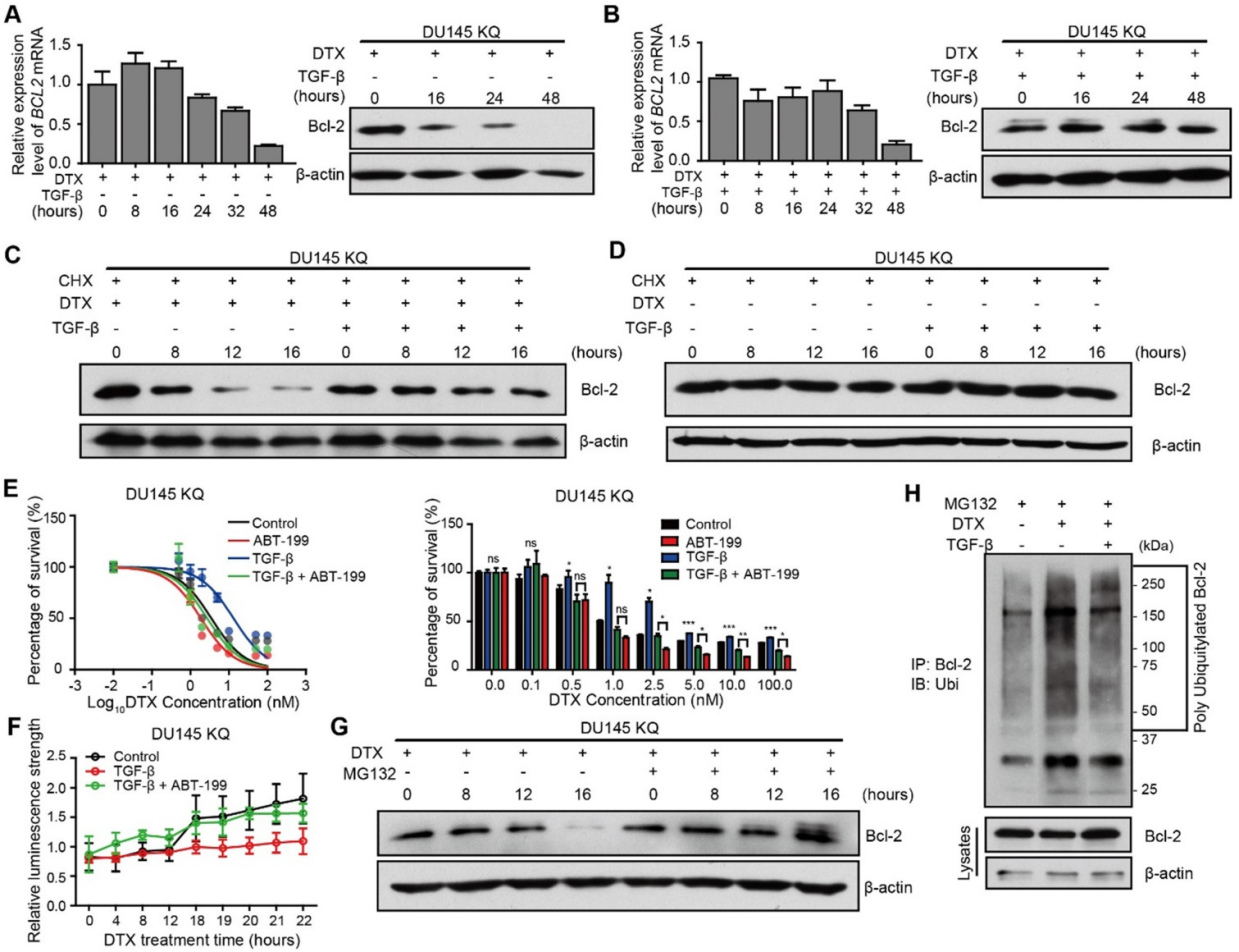
** TGF-β induces DTX resistance through stabilizing Bcl-2 protein.** (A, B) Detection of Bcl-2 mRNA and protein levels in DU 145 KQ cells treated with DTX (10 nM) alone (A), combination of TGF-β (10 ng/ml) and DTX (10 nM) (B) for different times as indicated by real-time qPCR (left panel) and Western blotting (middle panel) respectively. β-actin and GAPDH were used as internal controls for Western blotting and real-time qPCR, respectively. (C, D) Detection of Bcl-2 protein level by Western blotting in DU 145 KQ cells treated with different combinations for different times as indicated. DTX, 10 nM; TGF-β, 10 ng/μl; Cycloheximide (CHX), 10 µM. (E) Cytotoxicity assay measuring DTX resistance in DU 145 KQ cells after 72 hours of combined treatment with TGF-β (10ng/µl) and ABT-199 (1 µM). (F) Apoptosis and necrosis assay measuring DTX induced Annexin V staining in DU 145 KQ cells with combined treatment with TGF-β (10ng/µl) and ABT-199 (1 µM). (G) Western blotting analysis of Bcl-2 protein level in DU 145 KQ cells over 16 hours, MG-132 (10 µM) treatment 3 hours before protein collection. (H) Western blotting analysis of HA-tagged polyubiquitination in Bcl-2 antibody precipitated protein in 293T cells overexpressing Bcl-2 and HA-tagged ubiquitin. 16 hours of DTX (10 nM), TGF-β (10 ng/µl), 3 hours of MG-132 (10 µM) prior to protein collection. Cytotoxicity assay and real-time qPCR assay were performed in triplicate, and error bars represent the standard errors of the means. ns, *p* > 0.05; *, *p* ≤ 0.05; **, *p* ≤ 0.01; ***, *p* ≤ 0.001. DTX: docetaxel.

Inactivation of Bcl-2 is a recognized mechanism of apoptosis 43, 44, which is essential for DTX-induced cell death 45. We thus further investigated the role of Bcl-2 induced by the TGF-β/acetylated KLF5 axis in DTX resistance. Western blotting and quantitative RT-PCR were used to measure Bcl-2 protein level and mRNA level, respectively, after DTX treatment with or without TGF-β. We observed that 10 nM DTX treatment slightly upregulated *BCL2* mRNA level at 8 and 16 hours of treatment, and then downregulated it starting from 24 hours of treatment; however, the protein level was decreased starting from 16 hours of treatment (Figure 5A), which is ahead of the decrease in *BCL2* mRNA level, suggesting that the effects of DTX on Bcl-2 protein could be attributed to reason other than transcriptional regulation. Surprisingly, the combined treatment of TGF-β and DTX failed to decrease Bcl-2 protein level, although a lower mRNA level was observed starting from 8 hours of combined treatment (Figure 5B).

To further test if the increased protein level in the TGF-β and DTX combined treatment group was due to TGF-β's ability to inhibit degradation, we used cycloheximide (CHX) to block protein synthesis in KQ cells. Under protein synthesis blockade by CHX, DTX treatment decreased Bcl-2 protein level starting from 8 hours in DU 145 KQ cells, and combined treatment with TGF-β maintained the Bcl-2 protein level (Figure 5C). This further demonstrates that DTX led to Bcl-2 degradation and TGF-β can inhibit the degradation induced by DTX. At the same time, the Bcl-2 protein level was not affected by CHX alone or combined with TGF-β (Figure 5D). TGF-β did not affect Bcl-2 protein level; therefore, the aforementioned decrease in Bcl-2 protein level under TGF-β treatment is not due to degradation. On the other hand, although KR cells have much lower Bcl-2 level, Bcl-2 was downregulated by DTX, but maintained by treatment with TGF-β alone or the DTX and TGF-β combination in a pattern similar to KQ (Figure S4A-B). This result further suggests that TGF-β stabilized Bcl-2 under DTX treatment in an acetylated KLF5 independent manner.

Given the Bcl-2 stabilizing role of TGF-β under DTX treatment, we wondered if inhibition of Bcl-2 suppresses DTX resistance induced by TGF-β. As shown in Figure 2C-D, 10 ng/μl TGF-β treatment increased the DTX IC_50_ 2-fold in KQ cells. Interestingly, the addition of 1000 nM ABT-199 abolished the increase in IC_50_ induced by TGF-β treatment (Figure 5E). In the early apoptosis detection assay, instead of a sharp increase of Annexin V signal at 18 hours under DTX treatment, TGF-β treatment attenuated the upregulation of Annexin V signal. However, the addition of ABT-199 restored the induction of Annexin V signal by TGF-β (Figure 5F). We further measured the expression of PARP and its cleaved form in KQ cells via Western blotting, and found that DTX treatment at 10 nM for 20 hours induced PARP cleavage (Figure S5A). However, when the cells were co-treated with DTX and TGF-β, DTX showed weaker effect on PARP cleavage (Figure S5A). Interestingly, combined treatment of DTX, TGF-β and ABT-199 successfully rescued the downregulation of cleaved PARP by TGF-β (Figure S5A). Furthermore, flow cytometry detected early apoptosis signal induced after DTX treatment in KQ cells of DU 145 (Figure S5B). With the Annexin V+/PI- cell percentage as an indicator of early apoptosis, we observed that TGF-β alone decreased, while DTX alone increased, early apoptosis; DTX-induced early apoptosis was attenuated by TGF-β treatment; and addition of ABT-199 overcame the effect of TGF-β on DTX-induced early apoptosis (Figure S5B). These findings suggest that, as a potent Bc-2 selective inhibitor, ABT-199 sensitizes cells to DTX treatment by reversing the effect of TGF-β.

### TGF-β stabilizes Bcl-2 during DTX treatment via inhibition of ubiquitin-dependent protein degradation

Next, we aimed to identify the mechanism by which TGF-β inhibited DTX-induced Bcl-2 degradation. A thorough literature review reveals that Bcl-2 is degraded via ubiquitination-mediated proteasome degradation (Figure S4C). Therefore, we used 10 μM MG-132, a 26S proteasome inhibitor, to block proteasome-dependent degradation. MG-132 maintained the Bcl-2 protein level by reversing the downregulating effect of DTX (Figure 5G, S4D). This finding indicates that protein degradation by the 26S proteasome is the major process mediating Bcl-2 degradation. Furthermore, we explored whether poly-ubiquitination mediated the proteasome-dependent degradation of Bcl-2. We first overexpressed HA-tagged ubiquitin and Bcl-2 in 293T cells, then performed an immunoprecipitation assay to pull down Bcl-2 after 3 hours of MG-132 treatment. HA-tagged ubiquitination was detected in Western blotting assay with HA-tag antibody. DTX treatment increased the poly-ubiquitination of Bcl2 (Figure 5H, Lane 1 and 2), and this was reversed by treatment with the combination of TGF-β and DTX (Figure 5H, Lane 2 and 3). Together, DTX induced poly-ubiquitination of Bcl-2 and proteasome degradation, and TGF-β stabilized Bcl-2 via inhibition of Bcl-2 poly-ubiquitination.

### TGF-β/acetylated KLF5 signaling positively associates with Bcl-2 in prostate cancer patients

We established two DTX-resistant DU 145 cell lines, DU 145 DTX Resistant 50 (DDR50) and DU 145 DTX Resistant 100 (DDR100) by selection under increasing DTX concentration. DDR50 and DDR100 survived and grew in 50 nM and 100 nM DTX containing medium, respectively. Interestingly, Western blotting showed that both resistant cell lines expressed a higher level of KLF5, acetylated KLF5 and Bcl-2 (Figure S6A). Treatment with 500 nM ABT-199 significantly reduced cell survival with DTX treatment at 50, 100, 500, 1000 nM (Figure S6B). In addition, siRNA-mediated *KLF5* silencing in DDR50 cells sensitized them to DTX treatment by inhibiting cell survival with DTX treatment at 100, 500, and 1000 nM (Figure S6C). Therefore, Bcl-2 mediated DTX resistance, and the Bcl-2 inhibitor ABT-199 significantly sensitized DDR50 cells to DTX treatment. These data also suggest that acetylated KLF5/Bcl-2 signaling could be a fundamental mechanism mediating DTX resistance.

Prostate cancer patient data from two datasets was used to identify the roles of TGF-β, Bcl-2 and KLF5 in prostate cancer. First, we analyzed mRNA and protein level of BCL2 in the TCGA provisional prostate adenocarcinoma dataset with clinical information. Among the 499 patient samples, there were 187 stage I/II prostate cancer patient samples, 304 stage III and higher prostate cancer patient samples, and 8 missing values. In addition, there were 345 samples without lymph node metastasis, 80 with lymph node metastasis, and 74 missing values. There were 458 samples without distant metastasis (M0), 1 sample with distant metastasis (M1) and 40 missing values. Moreover, there were 292 samples with a Gleason score lower or equal to 7, 206 with a Gleason score higher than 7, and 1 missing value. We found that higher BCL2 mRNA and protein level were associated with older age, higher tumor stage, and higher Gleason score (Table 1).

**Figure 6:**
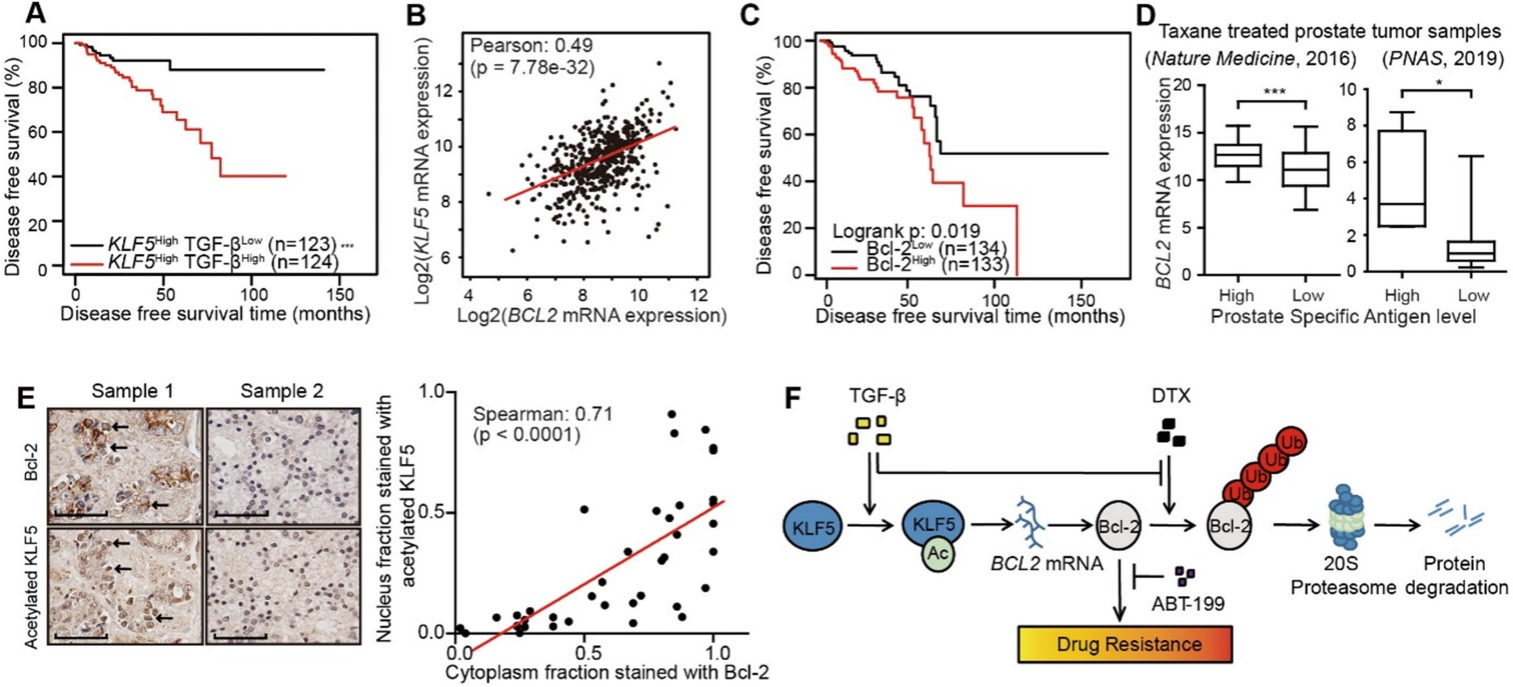
** Higher acetylated KLF5 correlates with higher Bcl-2 expression, and the latter associates with poorer survival of prostate cancer patients.** (A) Kaplan-Meier estimates of disease free survival in 247 advanced stage prostate cancer patients with *KLF5* mRNA level greater than median (TCGA, Provisional). (B) mRNA expression analysis and correlation of *BCL2* and *KLF5* from 499 prostate cancer patient samples (TCGA, Provisional). (C) Kaplan-Meier estimates of disease-free survival in 267 patients with prostate cancer (TCGA, Provisional). Bcl-2^low^: protein expression z-score less than mean. Bcl-2^high^: protein expression z-score greater than mean. (D) *BCL2* mRNA expression level in taxane treated prostate cancer patients with high and low prostate specific antigen (PSA) level. (E) Prostate cancer tissue arrays stained for acetylated KLF5 and Bcl-2 pictured to show correlation of acetylated KLF5 and Bcl-2. Two representative tumor samples are shown. (F) A schematic model shows that TGF-β and acetylated KLF5 signaling axis induce DTX resistance through Bcl-2 upregulation and stabilization. Error bars represent the standard errors of the means. ns, *p* > 0.05; *, *p* ≤ 0.05; **, *p* ≤ 0.01; ***, *p* ≤ 0.001. Scale bars, 100 µm. Magnification, X40. DTX: docetaxel.

Furthermore, we performed survival analysis in prostate cancer patient conditioned on TGF-β signaling and *KLF5* mRNA status. We defined a high level of TGF-β signaling as greater than median mRNA levels of *TGFB1* and either *TGFBR1* or *TGFBR2*. We found a high level of TGF-β signal was associated with a shortened disease free survival time (log-rank *p*-value = 0.018) (Figure S7A). Next, we performed survival analysis stratified on KLF5 mRNA level and TGF-β signal strength. In the 247 patients with* KLF5* mRNA level greater than median, a higher TGF-β signal correlated with significantly shorter disease free survival time (log-rank *p*-value < 0.0001) (Figure 6A). In contrast, in patients with lower KLF5 mRNA level, TGF-β signal did not correlate with significant survival changes (log-rank e*p*-values > 0.05). We further performed similar Kapan-Meier analysis in a 57 patient cohort 46 in which all patients had metastatic CRPC (mCRPC). We found that high level TGF-β was not associated with overall survival time (log-rank *p*-value = 0.516) (Figure S7B). However, in the 43 patients with* KLF5* mRNA level greater than first quartile, high TGF-β signal was associated with significantly shorter overall survival time (log-rank *p*-value = 0.019) (Figure S7C). This shows that TGF-β reduces overall survival time in an mCRPC patient group with high level of *KLF5* mRNA. In short, based on the findings in two prostate cancer patient datasets, a high level of TGF-β induced a shorter survival time in patients with high *KLF5* mRNA level.

In the TCGA provisional prostate adenocarcinoma dataset,* KLF5* mRNA levels co-expressed with that of *BCL2* with Pearson co-efficient value equal to 0.49 (*p*-value < 0.0001), which indicates an intermediate correlation (Figure 6B). Disease free survival data obtained from the same cohort found that higher level of Bcl-2 protein correlated with significantly shorter disease free survival time (log-rank *p*-value = 0.02) (Figure 6C). Moreover, in two individual prostate cancer studies 46, 47, we found that in patients with previous taxane treatment, higher levels of prostate specific antigen (greater than median) correlated with a higher level of *BCL2* mRNA (Figure 6D). In a prostate cancer tissue microarray, we performed immunohistochemistry staining of Bcl-2 and acetylated KLF5. We found the nuclear fraction stained with acetylated KLF5 co-localized and strongly correlated with the cytoplasmic fraction stained with Bcl-2 (Spearman co-efficient = 0.71, *p*-value < 0.0001) (Figure 6E). These findings further show that Bcl-2 and KLF5 play pivotal roles in prostate cancer progression not only *in vitro*, but also in clinical prostate cancer samples.

## Discussion

This study has revealed mechanisms that mediate DTX resistance in advanced prostate cancer (Figure 6F). Based on previous reports that TGF-β induces acetylation on KLF5 lysine 369 in HaCaT cells 24, 25 and prostate cancer cells 26, we found by *in vitro* and *in vivo* studies that TGF-β induced DTX resistance through the acetylation of KLF5. Additionally, we uncovered the function of TGF-β and acetylated KLF5 in Bcl-2 transcriptional regulation and stabilization. Using multiple patient datasets with expression profiles and clinicopathological information, we also found that TGF-β and higher KLF5 expression correlated with poor prostate cancer patient survival; and showed that BCL2 was a potential biomarker with both prognostic and predictive functions. Lastly, we found that inhibition of Bcl-2 by ABT-199 overcomes DTX resistance.

Drug resistance can lead to untreatable and lethal malignancies, and thus has been investigated extensively. Among various resistance mechanisms, EMT induced by modulation of the tumor microenvironment is a prominent contributor to drug resistance 13, mainly through cancer stem cell properties 48-50 and prolonged cell cycle 51. Notably, EMT, as a process critical for invasive and migratory phenotypes, can be activated by paracrine and autocrine TGF-β signaling 52, 53. Moreover, TGF-β induces taxane family drug resistance in various types of cancer including prostate cancer 5, 17. In addition to its tumor promoting properties, TGF-β is also known as a tumor suppressor due to its ability to inhibit cell proliferation 54, 55 and induce cell death 56, 57. Similarly, in the present study, we identified a novel mechanism of TGF-β dual function regulation. We found that DTX treatment acts as a “switch” for TGF-β dual function, and Bcl-2 is a critical factor to “turn on” the adaptive resistance promoting function of TGF-β. In the absence of DTX, TGF-β inhibits cell apoptosis by intrinsic mechanism in which transcriptionally activation Bcl-2 was regulated via KLF5. In contrast, in the context of DTX treatment, TGF-β inhibits cell apoptosis by stabilizing Bcl-2.

TGF-β is a ubiquitously-expressed cytokine that exhibits dual function in various biological processes. Long term, high dose inhibition of the TGF-β pathway may induce increased expression of oncoproteins that were sequestered by TGF-β in the initial stages of tumor formation 58. Indeed, a recent clinical trial evaluating the effects of TGF-β blockade by GC-1008 in metastatic breast cancer (clinical trial ID NCT01401062) has shown that blocking TGF-β alone is insufficient in controlling tumor growth even when combined with radiation 59. Therefore, targeting the TGF-β signaling pathway for therapeutic benefit requires consideration of multimodal therapeutic strategies and biomarkers for stratification of patients based on predicted response.

Our findings suggest that use of one or more currently available agents targeting the TGF-β/acetylated KLF5/BCL2 signaling axis is beneficial to patients with DTX-resistant prostate cancer. We demonstrated that TGF-β and Bcl-2 had potential prognostic values in prostate cancer patients (Figure 6A, C, S7A-C) and that Bcl-2 inhibitor, ABT-199, blocked DTX resistance *in vitro* mediated by TGF-β (Figure 5E) and acetylated KLF5 mediated DTX resistance *in vitro* (Figure 3F), which warrant *in vivo* studies to test TGF-β receptor 1 inhibitor (e.g., SB-505124) and Bcl-2 inhibitor (e.g., ABT-199) for their therapeutic value in overcoming DTX resistance. Interestingly, a recent epidemiological study has showed that, naftopidil, a naphthalene-based α1-adrenoceptor antagonist, reduces prostate cancer incidence due to its blocking effect on TGF-β signaling and Bcl-2 expression 60, which is consistent with our finding that lower levels of TGF-β signaling and Bcl-2 expression correlated with prolonged disease free survival in patients with advanced prostate cancer. Therefore, as a single agent inhibiting TGF-β signaling and Bcl-2 expression, naftopidil could be an effective inhibitor of the TGF-β/acetylated KLF5 signaling axis for overcoming DTX resistance in prostate cancer.

Our findings further suggest that KLF5 acetylation could be an alternative drug target for overcoming DTX resistance in prostate cancer. In the absence of acetylated KLF5, TGF-β was compromised in inducing DTX resistance. Considering that paracrine TGF-β is present in the tumor microenvironment, the finding from the xenograft mouse model further supports a critical role of acetylated KLF5 in DTX resistance, which thus suggests acetylated KLF5 as an alternative target of TGF-β's tumor promoter function. Notably, as a basic transcription factor, KLF5 regulates a broad spectrum of biological processes including cell cycle progression, migration, and differentiation 61; and inhibition of KLF5 function may induce significant off-target effect 62, 63. Therefore, KLF5 acetylation could be an alternative drug target that is more specific for inhibiting KLF5 function in drug resistance.

Furthermore, our study identified several candidate biomarkers for advanced prostate cancer. Analysis of 499 prostate cancer samples and 57 mCRPC samples demonstrated that higher TGF-β signaling activity is prognostic of shorter overall survival in patients with higher *KLF5* mRNA levels. With high levels of KLF5, TGF-β could exert its function through acetylation of KLF5. In addition, higher Bcl-2 protein level was also prognostic of worse disease free survival. Analysis of two datasets of mCRPC patients showed that BCL2 expression could potentially predict treatment responses in prostate cancer patients, as in patients with the history of taxane treatment, higher BCL2 levels correlated with an elevated prostate specific antigen (PSA) level, which is the indicator of prostate cancer biochemical recurrence 64. The bone is a major metastasis site of advanced prostate cancer, and enriched TGF-β in the bone promotes bone destruction and metastasis 65. It is thus possible that BCL2 expression in primary tumor and bone metastasis could be predictive of taxane treatment response.

In summary, this study has revealed that TGF-β induced DTX resistance in both intrinsic and adaptive pathways. Intrinsically, TGF-β acts through the acetylation of KLF5 to transcriptionally upregulate Bcl-2. Adaptively, with the presence of DTX, TGF-β stabilizes Bcl-2 through ubiquitination to mediate DTX resistance. Inhibition of Bcl2 by ABT-199 overcame DTX resistance. Accordingly, these results suggest that the TGF-β/acetylated KLF5/BCL2 signaling axis mediates DTX resistance in prostate cancer and that targeting this signaling axis might be a novel therapeutic approach for the treatment of chemo-resistant prostate cancer.

## Supplementary Material

Supplementary figures.

## Figures and Tables

**Table 1:** Association of BCL2 Expression with Clinical and Pathologic Variables in 499 Primary Tumors from Prostate Cancer

		BCL2 mRNA expression (M/Y)					Bcl-2 protein expression (M/Y)		
Variable	Total Cases	Lower	Higher	*P* value (M/Y)*		Total Cases	Lower	Higher	*P* value (M/Y)*
Age (years)									
<61	223	115/49	108/174	0.428/0.009 #		139	50/50	89/89	0.002/0.002
>=61	275	132/36	143/239			126	60/60	66/66	
Stage									
I/II	187	101/116	86/71	0.198/0.035 #		85	49/40	36/45	0.009/0.003
III/IV	304	146/159	158/145			180	83/59	97/121	
Lymph node									
-	345	170/267	175/78	0.626/0.163		156	97/87	59/69	0.768/0.032 #
+	80	37/56	43/24			38	24/14	14/24	
Gleason score									
<=7	292	149/238	143/54	0.585/0.005 #		147	86/94	61/53	0.001/0.000
>7	206	100/146	106/60			118	46/50	72/68	

Data are given as number of tumors. Higher or lower BCL2 expression was relative to the median (M) of 409.17 (mRNA, TPM) and -0.13 (p, z-scores) or the optimal cutoff point determined by the Youden Index (Y) in tumors, which was 195.27 (m) and -0.13 (p) for age, 439.35 (m) and -0.39 (p) for tumor stage, 657.03 (m) and -0.10 (p) for lymph node status, 656.28 (m) and -0.03 (p) for gleason score status. * *P* values were determined by using the Pearson X2 test. # The *P* value became smaller than 0.05 after the optimal cutoff point determined by the Youden Index was applied.

## Abbreviations

CRPC: castration-resistant prostate cancer
DDR50: DTX-resistant cell lines tolerated a final DTX concentration of 50 nM
DDR100: DTX-resistant cell lines tolerated a final DTX concentration of 100 nM
DTX: docetaxel
EV: empty vector
KBE: KLF5 binding element
KLF5: Krüppel-like factor 5
KR: acetylation deficient KLF5 mutant (KLF5^K369R^)
KQ: acetylation mimicking KLF5 mutant (KLF5^K369Q^)
SB: SB-505124
TCGA: The Cancer Genome Atlas
