# Supplementary material for: TGF-β causes Docetaxel resistance in Prostate Cancer via the induction of Bcl-2 by acetylated KLF5 and Protein Stabilization
Source: Theranostics. 2020 Jun 18;10(17):7656–70. doi: 10.7150/thno.44567 (PMC7359077; doi:10.7150/thno.44567)
Supplement: Supplementary file 1 — Supplementary figures. [file thnov10p7656s1.pdf]

Supplementary Materials

TGF-β causes docetaxel resistance in prostate cancer via the induction of Bcl-2 by acetylated KLF5 and protein stabilization

Li et al.

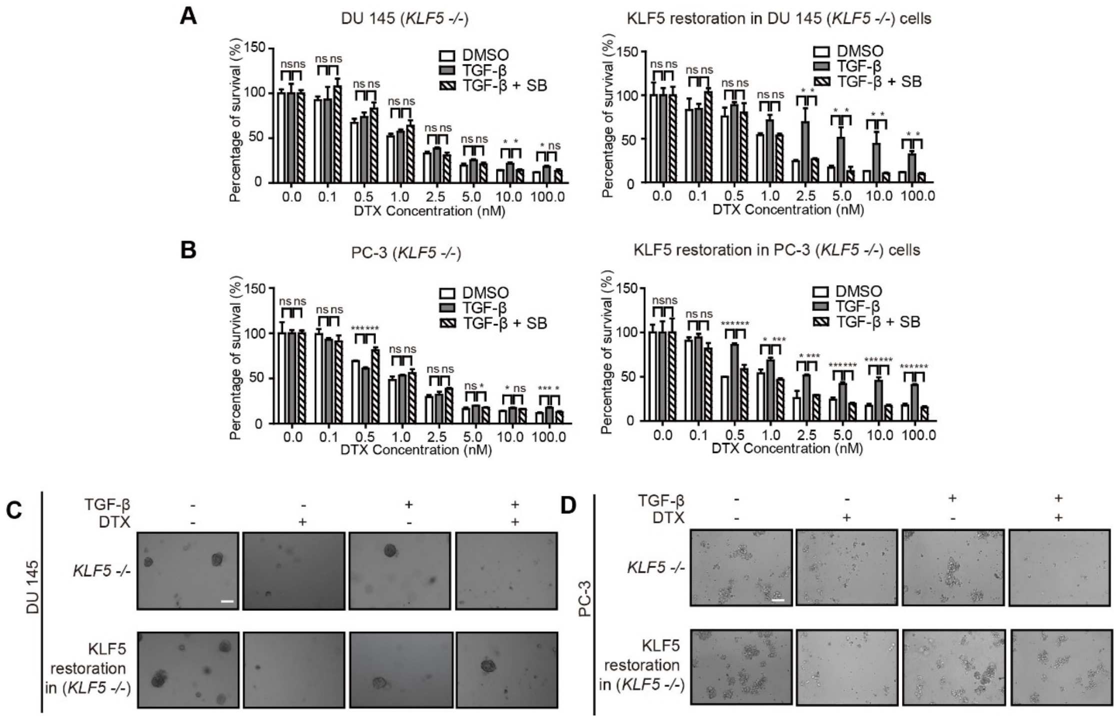

Figure S1. KLF5 is required by TGF-β to induce DTX resistance in prostate cancer cells. (A, B) Cytotoxicity assay in DU 145 and PC-3 cell variants with concomitant treatments of DTX and TGF-β1 (10 ng/µl) and/or SB505124 (2.5 µM). *KLF5 -/-*, endogenous *KLF5* was knocked out. (C, D) Colony formation assay of *KLF5 -/-* DU 145 and PC-3 cells with or without wild type *KLF5* restoration in Matrigel treated with DTX (1 nM) and/or TGF-β1 (10 ng/µl). Matrigel colony formation assay was performed in duplicate, and error bars represent the standard errors of the means. ns, $p > 0.05$; *, $p \leqslant 0.05$; **, $p \leqslant 0.01$; ***, $p \leqslant 0.001$. Scale bars, 100 µm. Magnification, X10. DTX: docetaxel; SB: SB-505124.

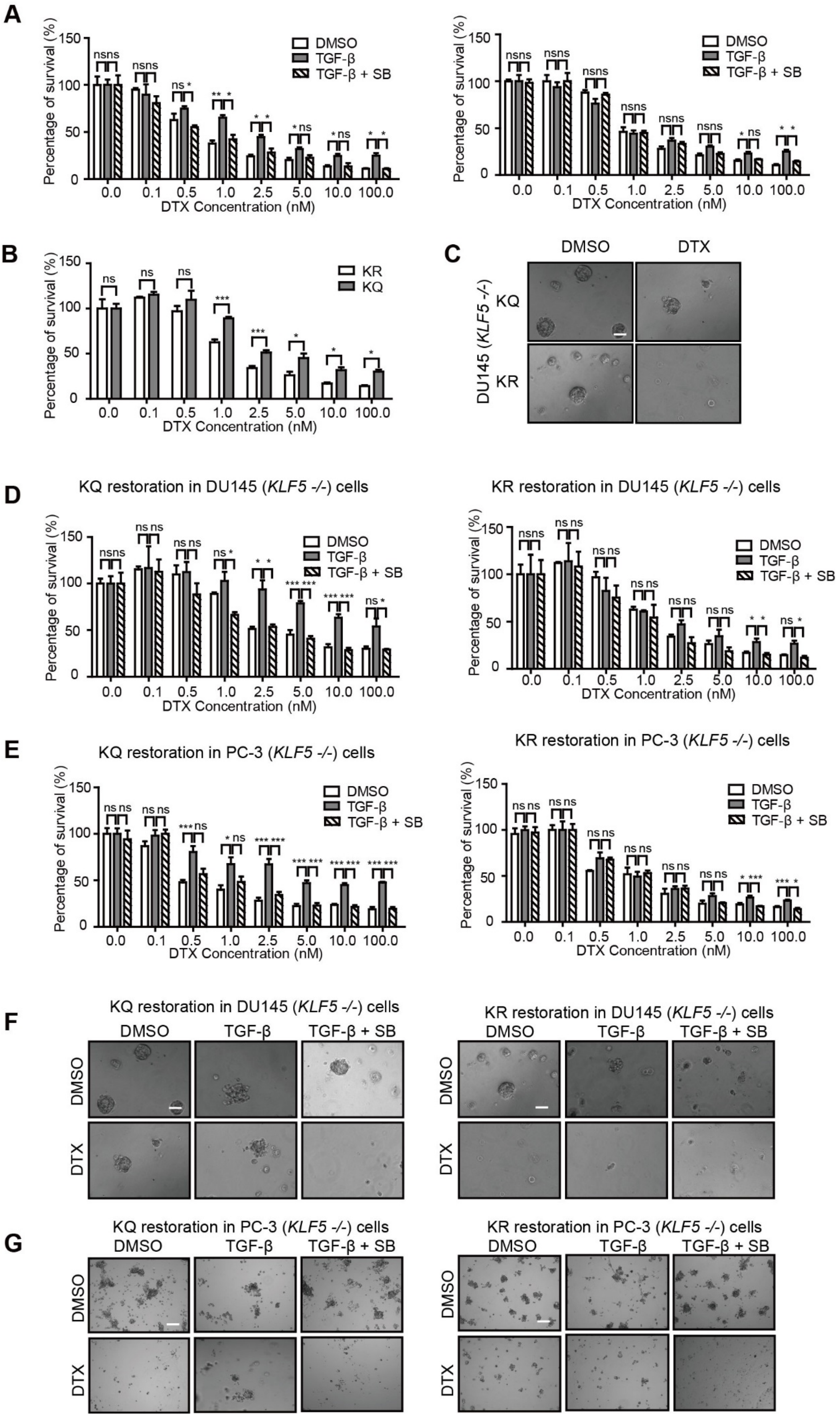
A
Percentage of survival (%)
DMSO
TGF-β
TGF-β + SB
DTX Concentration (nM)
0.0
0.1
0.5
1.0
2.5
5.0
10.0
100.0
B
KR
KQ
C
DMSO
DTX
DU145 (KLF5 -/-)
KQ
KR
D
KQ restoration in DU145 (KLF5 -/-) cells
KR restoration in DU145 (KLF5 -/-) cells
E
KQ restoration in PC-3 (KLF5 -/-) cells
KR restoration in PC-3 (KLF5 -/-) cells
F
KQ restoration in DU145 (KLF5 -/-) cells
KR restoration in DU145 (KLF5 -/-) cells
G
KQ restoration in PC-3 (KLF5 -/-) cells
KR restoration in PC-3 (KLF5 -/-) cells

Figure S2. KLF5 acetylation at K369 mediates DTX resistance in prostate cancer cells. (A) Cytotoxicity assay in DU 145 (*KLF5*-/-) cells expressing wild type *KLF5* and acetylation deficient mutant KLF5^{K369R} (KR) with concomitant treatments of DTX and TGF-β1 (10 ng/μl) and/or SB505124 (2.5 μM). (B, C) Cytotoxicity assay and colony formation assay in Matrigel of DU 145 (KLF5-/-) cells expressing KR and acetylation mimicking mutant KLF5^{K369Q} (KQ) treated with DTX (1 nM). (D - G) Cytotoxicity assay of DTX (D, E) and colony formation assay with 1 nM DTX (F, G) in DU 145 and PC-3 (KLF5-/-) cells expressing KR and KQ with concomitant treatments of TGF-β1 (10 ng/μl) and/or SB505124 (2.5 μM). Cytotoxicity assay and Matrigel colony formation assay were performed in triplicate, and error bars represent the standard errors of the means. ns, $p > 0.05$; *, $p \leqslant 0.05$; **, $p \leqslant 0.01$; ***, $p \leqslant 0.001$. Scale bars, 100 μm. Magnification, X10. DTX: docetaxel; SB: SB-505124.

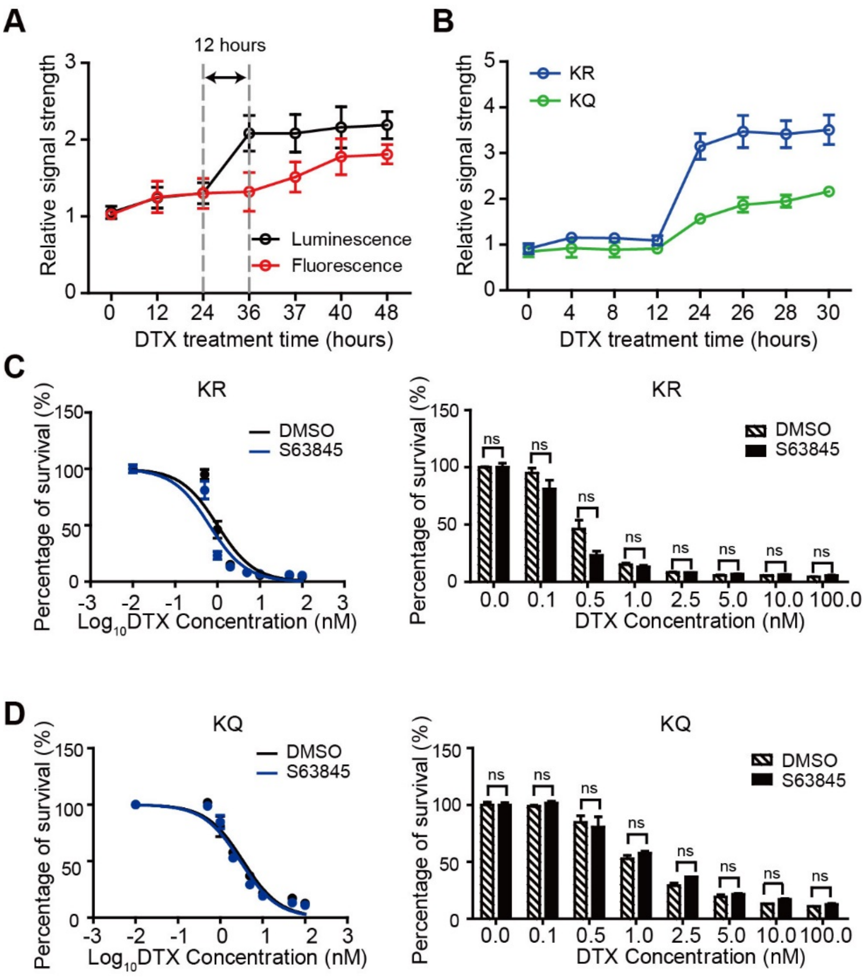

Figure S3. DTX is less effective in inducing early apoptosis in cells expressing acetylated KLF5, and Mcl-1 did not play an apparent role in TGF-β induced DTX resistance in KQ cells. (A, B) Apoptosis and necrosis assays were used to measure early apoptosis response in DU 145 parental cells (A) and KR and KQ cells (B) with DTX treatment (10 nM). (C, D) Cytotoxicity assay of DTX-treated DU 145 (KLF5-/-) cells expressing KR and KQ with or without concomitant treatment of S63845 (1 μM). Cytotoxicity assay was performed in triplicate, and error bars represent the standard errors of the means. ns, $p > 0.05$; *, $p \leqslant 0.05$; **, $p \leqslant 0.01$; ***, $p \leqslant 0.001$. DTX: docetaxel.

A

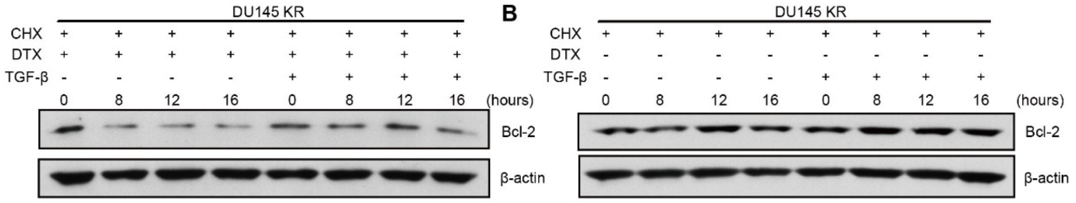

C

Mechanisms Regulating Bcl-2 Stablity.

Function	Regulation Type	Findings	Referrence
Stablize	S-nitrosylation	In H460 cells, nitric oxide induced S-nitrosylation on cystein residues.	Chanvorachote et al., 2006
		In H460 cells, Cys158 and Cys229 of Bcl-2 were nitrosylated.	Azade et al., 2006
	Phosphorylation	In HeLa cells,the MAP kinase sites Thr74 and Ser87 of Bcl-2 are phosphorylated by MAP kinase.	Breitschopf et al., 2000
	Mono-ubiquitination	In 293 cells, Parkin induced monoubiquitination on C-terminus of Bcl-2.	Chen et al., 2010
Destablize	S-denitrosylation	In HeLa, A375, and 7860, MDA-7/IL24 induced s-denitrosylation on Bcl-2.	Tian et al., 2012
	Dephosphorylation	In HUVEC cells, TNF-alpha induced dephosphorylation on Thr 56, Thr 74, and Ser 87 (major role) of Bcl-2.	Dimmeler et al., 1999
		In H460 cells , Cisplatin/hydrogen peroxide/hydroxyl radical induced dephosphorylation of Bcl-2.	Wang et al., 2008<br>Luanpotpong et al., 2011
	Poly-ubiquitination	In H460 cells, Cr(VI)-I induced ROS led to polyubiquintinated Bcl-2.	Azad et al., 2008
		In SW480, PPAR-alpha induced polyubiquintination on Lysine 22 with Bcl-2.	Gao et al., 2015
		In HeLa and MEF, lysine 17 of Bcl-2 was ubiquintinated by E3 ligase XIAP.	Edison et al., 2017
		In HEK293, Keap1:Cul3-Rbx1 induced polyubiquintnation on lysine 17.	Niture et al., 2011
		In 293T, FBXO10 polyubiquintinated Bcl-2.	Chiorazzi et al., 2013
	Caspases Cleavage	In Huh7, caspase 3 (major role) and caspase 9 cleaved Bcl-2, FKBP38 prevented the cleavage by binding to flexible loop domain of Bcl-2.	Choi et al., 2010

D

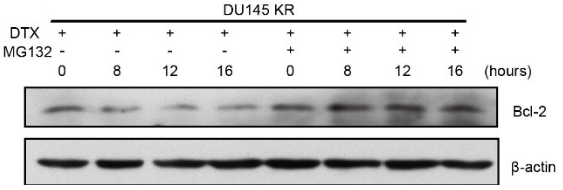

Figure S4. TGF-β induces DTX resistance by stabilizing Bcl-2 protein in DU 145 cells expressing acetylation-deficient KLF5. (A, B) Detection of Bcl-2 protein level by Western blotting in DU 145 KR cells treated with different combinations of DTX (10 nM), TGF-β (10 ng/μl), and cycloheximide (CHX, 10 μM) for indicated time. (C) A literature review of molecular mechanisms that regulate Bcl-2 stability. (D) Western blotting analysis of Bcl-2 protein in DU 145 KR cells after 16 hours of DTX treatment. MG-132 treatment was applied at 10 μM for 3 hours before protein collection. Cytotoxicity assays were performed in triplicate, and error bars represent the standard errors of the means. ns, $p > 0.05$; *, $p \leqslant 0.05$; **, $p \leqslant 0.01$; ***, $p \leqslant 0.001$. DTX: docetaxel.

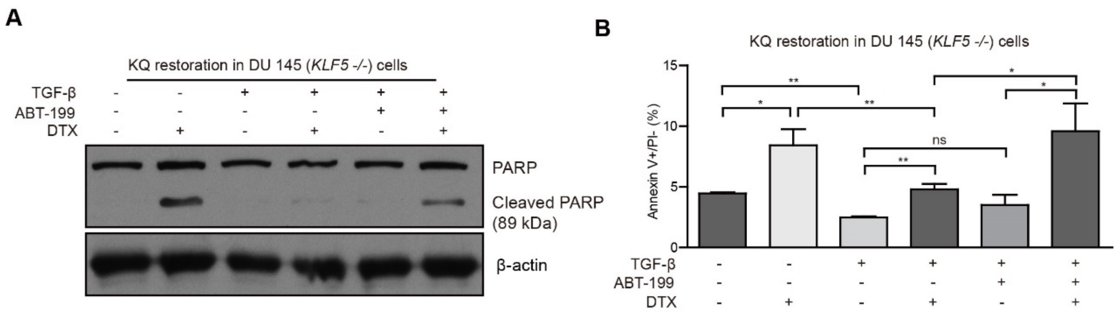

Figure S5. TGF-β induces DTX resistance through apoptosis inhibition. Detection of PARP and cleaved PARP protein levels by Western blotting (A) and Annexin V +/PI – cell percentage by flow cytometry analysis (B) in DU 145 KQ cells treated with different combinations of DTX (10 nM), TGF-β (10 ng/μl), and ABT-199 (1000 μM) for 20 (A) or 16 hours (B). Flow cytometry analysis was performed in triplicate, and error bars represent the standard errors of the means. ns, $p > 0.05$; *, $p \leqslant 0.05$; **, $p \leqslant 0.01$; ***, $p \leqslant 0.001$. DTX: docetaxel.

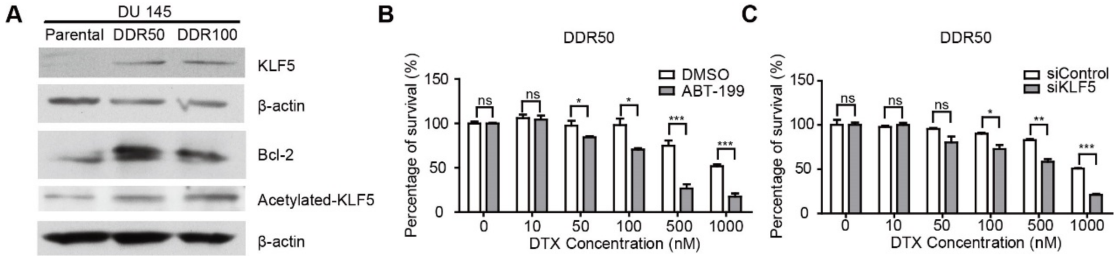

Figure S6. Bcl-2 mediates DTX resistance in prostate cancer cells. (A, B) Detection of KLF5, Bcl-2, and acetylated-KLF5 in parental cells and Docetaxel Resistant 50 and 100 (DDR50 and DDR100) cells of the DU 145 cell line. (B) Cytotoxicity assay of DTX in DDR50 cells with or without ABT-199 treatment (500 nM). (C) Cytotoxicity assay of DTX in DDR50 cells with or without *KLF5* silencing by siRNA. Cytotoxicity assays were performed in triplicate, and error bars represent the standard errors of the means. ns, $p > 0.05$; *, $p \leqslant 0.05$; **, $p \leqslant 0.01$; ***, $p \leqslant 0.001$. DDR50: DTX-resistant cell lines tolerated a final DTX concentration of 50 nM;

DDR100: DTX-resistant cell lines tolerated a final DTX concentration of 100 nM.

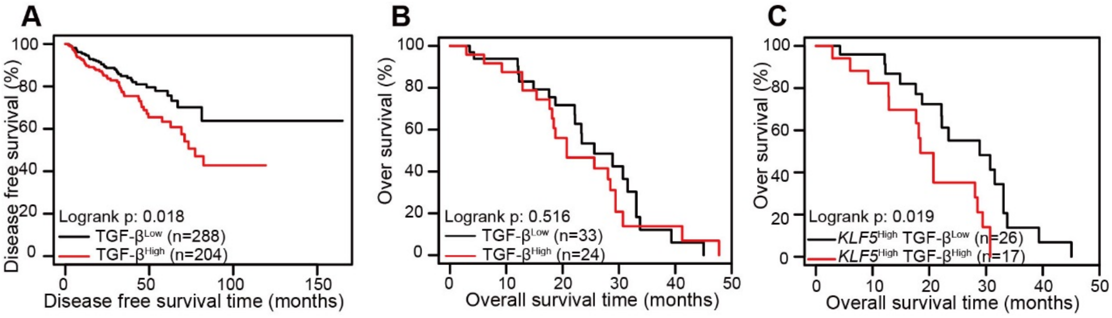

Figure S7. Higher TGF-β signaling activity and higher *KLF5* mRNA level correlate with poorer survival of prostate cancer patients. (A) Kaplan-Meier estimates of disease free survival in 492 patients with advanced prostate cancer (TCGA, Provisional). (B, C) Kaplan-Meier estimates of overall survival in 57 (B) and 43 (C) patients with castration-resistant prostate cancer. *KLF5*^{low}, mRNA expression z-score less than median; *KLF5*^{high}, mRNA expression z-score greater than median, TGF-β^{high}, TGFB1 and either TGFBR1 or TGFBR2 greater than median; and TGF-β^{low}, TGFB1 and either TGFBR1 or TGFBR2 equal to or smaller than median.
